# A Cellulose-Based Dual-Crosslinked Framework with Sensitive Shape and Color Changes in Acid/Alkaline Vapors

**DOI:** 10.3390/polym16111547

**Published:** 2024-05-30

**Authors:** Yuxin Sun, Xinye Qian, Yan Gou, Chunling Zheng, Fang Zhang

**Affiliations:** College of Food Science and Light Industry, Nanjing Tech University, Nanjing 211800, China; 202161119011@njtech.edu.cn (Y.S.);

**Keywords:** cellulose, dual-crosslink, deformation, color change, acid/alkaline vapors

## Abstract

Cellulose detectors, as green sensors, are some of the defensive mechanisms of plants which combat environmental stresses. However, extracted cellulose struggles to fulfil these functionalities due to its rigid physical/chemical properties. In this study, a novel cellulose dual-crosslinked framework (CDCF) is proposed. This comprises a denser temporary physical crosslinking bond (hydrogen bonding) and a looser covalent crosslinking bond (N,N-methylenebisacrylamide), which create deformable spaces between the two crosslinking sites. Abundant pH-sensitive carboxyl groups and ultralight, highly porous structures make CDCF response very sensitive in acid/alkaline vapor environments. Hence, a significant shrinkage of CDCF was observed following exposure to vapors. Moreover, a curcumin-incorporated CDCF exhibited dual shape and color changes when exposed to acid/alkaline vapors, demonstrating great potential for the multi-detection of acid/alkaline vapors.

## 1. Introduction

The effective identification of alkaline/acid vapors is indispensable for detecting toxic substances in substances used in industrial production or for everyday needs, such as acetic acid and ammonia vapors, which are deceptively hazardous to human health [1]. However, traditional detection methods are used to detect optical, electrical, thermal, magnetic, and other stimuli and transfer them into mechanical energy by visualizing their indicators [2,3,4,5,6]. This requires complicated technologies and expensive instruments, and thus application scenarios are often limited. Some plants have evolved mechanisms as stress responses to environmental changes, such as mimosa herb, which can bend its leaves when subjected to external stimuli, or triangle plum, which can change its color upon contact with formaldehyde. Observing these plants offered an intuitive way for ancient people to identify environmental stresses [7] and use natural materials to detect shape or color changes, thus perceiving environmental changes. 

Cellulose is one of the most abundant natural resources on Earth and is considered an ideal material for the preparation of observable detection materials due to its biocompatibility, renewability, biodegradability, thermal stability, chemical stability, and low cost [8,9,10]. Cellulose-based observable detection materials are organic–inorganic hybrids, which include inorganic–organic free-casting hybrid membranes, electro-spun nanomembranes, and porous hybrid detectors [11,12,13]. Cellulose nanofibers derived from rice husks are sensitive to water-soluble gases, such as ammonia and acetone [14]. Pongpat Sukhavattanakul et al. developed a sensor based on bacterial cellulose. The film can change color under hydrogen sulfide gas [15]. While most of these materials are associated with a single color change or flat curl. This raises the question of whether 3D deformable cellulose-based materials can be fabricated and used more extensively in a variety of environments. However, the low concentration of the relevant gas/vapor has limited the application of 3D materials in this field.

Nanocellulose foam, prepared by freeze-drying or supercritically drying crosslinked nanocellulose gels, could preserve porous and lightweight structures [16,17]. These structures offer an excellent platform for gas/vapor adsorption. However, the crosslinking of this foam leads to such a fixed network structure [18] that the challenge of shape changes in acid/alkaline vapors should be addressed. TEMPO-oxidized nanocellulose (CNF) endows carboxyl groups to the cellulose backbone [19]; therefore, the interactions between oxidized CNFs are constrained by pH values, since COO^−^ is strongly affected by ionic strength. The hydrogen bonding interaction between COOH and OH is strengthened under acidic conditions and broken down with higher pHs. Polyacrylate (PAA) is one of the most common pH-sensitive polyanions, and it contains abundant carboxyl groups [20]. We assumed that the incorporation of PAA would endow CNF foam with another crosslinking point and more carboxyl groups, thus making the polymer foam deformable in acid/alkaline vapors.

In this study, a novel CNF/PAA dual-crosslinked framework (CDCF) comprising denser temporary physical crosslinking and looser covalent crosslinking, which creates a deformable space between the two crosslinking sites, is proposed. Furthermore, an AA monomer was polymerized in situ and covalently crosslinked in the hydrogen-bonded CNF gels, causing a 12-fold increase in volume. After freeze-drying, CDCF becomes highly porous, very lightweight, and contains abundant pH-sensitive groups, and thus decreases remarkably in alkaline/acid vapors. More interestingly, curcumin-incorporated CDCF undergoes a noticeable color change during the decrease in volume.

## 2. Experimental Section

Nanocellulose (TEMPO-CNF, 1% wt) was purchased from ScienceK (Beijing, China). N,N’-Methylenebisacrylamide(BMA, A.R.) and span-85(A.R.) were purchased from Aladdin Biochemical Technology, Shanghai, China. Mineral oil (C.P.), acrylic acid (AA, A.R.), acetic acid (A.R.), and potassium persulfate (KSP, A.R.) were purchased from Shanghai Macklin Biochemical Technology, Shanghai, China. Curcumin (A.R.) was purchased from Shanghai Yuanye Bio-Technology Co., Ltd., Shanghai, China. Ammonium hydroxide (A.R.) was purchased from Shanghai Zhanyun Chemical Co., Shanghai, China. Hydrochloric acid (36–38% wt) was purchased from Sinopharm, Beijing, China. Ethanol (A.R.) was purchased from Wuxi Yasheng Chemical Co., Wuxi, China.

### 2.1. Fabrication of Nanocellulose Aerogels

A 1 wt% suspension of CNF was passed through a syringe (syringe capacity: 2.5 mL; needle diameter: 0.6 mm) and dropped into a 0.1 mol/L aqueous HCl solution under gentle stirring. Physically crosslinked gel spheres were obtained. Acrylic acid (5 wt%), BMA (0.25 wt%, 0.5 wt%, and 1 wt%), and potassium persulfate (2 wt%) were dissolved in pure water in a certain proportion and mixed well, and the CNF gel spheres were immersed in the prepared aqueous solution, gently stirred for 4 h, filtered, and then transferred to a three-necked flask. Liquid paraffin and emulsifier span-85 were added at a ratio of 1:1 to prevent crosslinking between the spheres. The CNF gel was heated to 60 °C in a water bath. After polymerization for 2 h, the gel was washed several times with excess ethanol and distilled water until it became neutral. The purified samples were then frozen with liquid nitrogen and dried with a freeze dryer for 24 h. The samples with an AA concentration of 5% and BMA concentrations of 0.25%, 0.5%, and 1% were named CDCF-1, CDCF-2, and CDCF-3.

### 2.2. Density and Porosity

The density and porosity of the prepared spherical aerogel specimens were determined. The mass of the aerogel (*m*_1_) was weighed, the diameter (*d*) of the aerogel was measured with an electronic digital ruler, and the volume (*V*) was calculated. The density of the aerogel was calculated according to Equation (1) [21]:

(1) $$\rho =\frac{m_{1}}{V}$$

The density of the solid materials was calculated according to Equation (2) [22] based on the solid density of each component and their weight ratios:

(2) $$\rho_{s}=\frac{1}{\frac{W_{CNF}}{\rho_{CNF}}+\frac{W_{AA}}{\rho_{AA}}}$$

where *W* is the weight percentage of the different components and *ρ_s_* is the solid density of the composite material. *ρ_CNF_* and *ρ_AA_* are the solid density values of CNF and AA: 1.46 and 1.05 g/cm^3^, respectively.

The pore fraction of aerogels was calculated according to Equation (3) [23]:

(3) $$P=\left(1-\frac{\rho }{\rho_{s}}\right)\times 100\%$$

where *ρ* is the density of the aerogel and *ρ_s_* is the density of the solid materials.

### 2.3. The Characterization of Aerogels

Scanning electron microscopy was used to observe the microscopic morphology of the aerogel spheres. A microblock sample was directly placed onto a conductive adhesive, and a sputter instrument (Quorum SC7620, Quorum Technologies, Laughton, UK) was used to spray gold for 45 s at 10 mA. Fourier-transform infrared spectroscopy was used to illustrate the changes in chemical structure. Spectra were recorded using a Thermo Scientific Nicolet iS20 (Thermo Scientific, Waltham, MA, USA) with a detector at 4 cm^−1^, a resolution ranging from 500 to 4000 cm^−1^, and 32 scans per sample. The elemental content contained in the cellulose aerogel material was determined using the XPS method. Samples of approximately 5 mm in size were cut and pasted onto the sample tray, and the samples were placed in the sample chamber of a Thermo Scientific K-Alpha XPS instrument. The samples were fed into the analytical chamber when the pressure in the sample chamber was less than 2.0 × 10^−7^ mbar, with a spot size of 400 μm, an operating voltage of 12 kV, and a filament current of 6 mA; the full-spectrum scanning flux energy was 150 eV, with a step size of 1 eV. The weight loss of each cellulose aerogel and its thermal stability were tested using a Netzsch STA 449 F5 (Selb, Germany) thermogravimetric analyzer by weighing about 10 mg of the sample, placing it in an oxidizing crucible, and heating it from 30 °C to 800 °C at an elevation rate of 20 °C/min, with the entire experiment being carried out under nitrogen protection.

### 2.4. Vapor Sensitivity Testing

An aerogel sphere with a diameter of about 1 cm was taken as a sample. Then, it was connected and fixed with 10 cm copper wires on both sides of the aerogel sphere, and a sensing test was carried out under the conditions of an initial potential of 0 V and a vapor flow rate of 0.1 L/min. Based on Henry’s law, we obtained ammonia and acetic acid gas with concentrations of 10, 20, 50, 100, 200, and 300 ppm. Subsequently, the sensitivity of the aerogel was tested under different vapor concentrations (ammonia and acetic acid) [24]. The diameter of the aerogels was measured using an electronic digital ruler when the concentrations of the ammonia and acetic acid vapors were between 10 and 300 ppm, and three parallel experiments were conducted for each group of samples. Additionally, photographs were taken to record the color changes of CDCF-1-CUR when placed in a concentration of 300 ppm ammonia and acetic acid vapor for 200 s. The time–current curve of the aerogel was plotted, and its vapor sensitivity could be calculated using the following Equation (4) [25]:

(4) $$S=\frac{I_{g}-I_{0}}{I_{0}}\times 100\%$$

where *S* is the vapor sensitivity of the aerogel, *I_g_* is the maximum current, and *I*_0_ is the equilibrium current.

## 3. Results and Discussion

**Figure 1:**
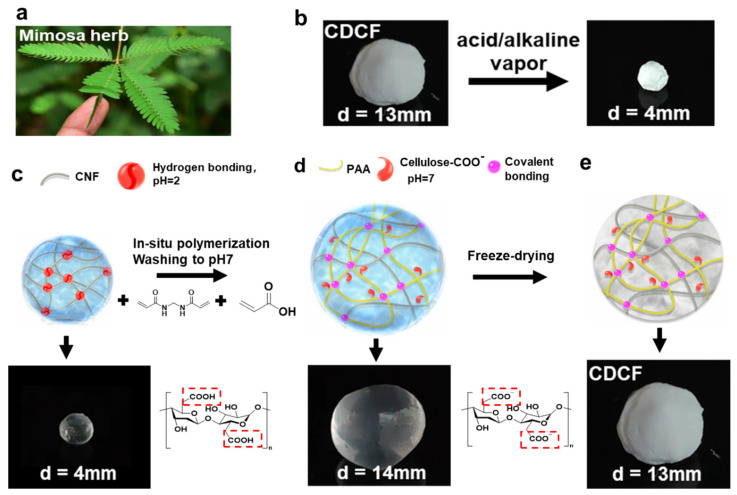
(**a**) Mimosa herb bends its leaflets when subjected to external stimuli. (**b**) The cellulose dual-crosslinked framework (CDCF) shrinks when immersed in acid/alkaline vapors. (**c**) CNF hydrogel bead generated from physical crosslinking at pH 2. (**d**) In situ polymerization of AANa/BMA in the hydrogen-crosslinked CNF gel sphere, which was then washed to pH = 7. (**e**) Highly porous CDCF was generated in the freeze-drying process of the synthesized gel.

Plants with special response mechanisms can avoid environmental danger by moving or changing colors—for example, mimosa herb retracts its leaves when subjected to external stimuli (Figure 1a)—and this inspired the development of shape-changing sensors. For this purpose, a cellulose double-crosslinked framework was prepared using free radical polymerizations of AA in situ, as well as BMA in CNF gels, as shown in Figure 1c–e (the procedure diagram is shown in Figure S1). A CNF gel was formed via a dropping process into HCl solution with pH = 2 using a syringe. Due to electrostatic interactions in the acid, the hydrogen bonding between -COOH and -OH was enhanced and gel spheres rapidly formed, resulting in dense and temporary hydrogen-bonded CNF gels (Figure 1c). Additionally, a CNF gel was absorbed by the AA monomers, the crosslinkers BMA and AA were polymerized in the CNF gel, and covalent crosslinking between PAA and CNF occurred. There are two roles for acrylic in CDCF. First, free radical polymerizations of AA and BMA form the covalent crosslinking, thus creating deformable spaces between the two crosslinking sites (hydrogen bonding and covalent bonding). Secondly, the carboxyl groups in the PAA chains impart pH sensitivity to CDCF as well. After the samples were washed until they were neutral, the hydrogen bonds separated, and thus the ball expanded from 4 to 14 mm. In this situation, covalent bonding maintained a gel structure (Figure 1d). The expanded gel ball was then freeze-dried to CDCF (Figure 1e). The size of the CDCF was slightly decreased due to vacuum suction [26].

**Figure 2:**
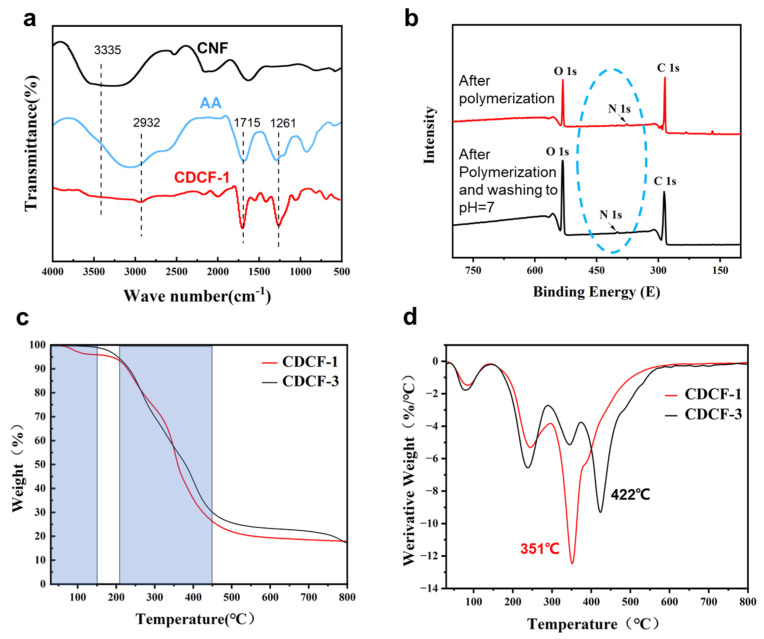
(**a**) FTIR curves of CNF, AA, and CDCF. (**b**) XPS curves of CDCF gel before and after the washing treatment. (**c**,**d**) TGA and DTG curves of CDCF-1 and CDCF-3.

Chemical groups were monitored using FTIR to verify the synthesis of CDCF. Figure 2 shows the FTIR spectra of CNF, AA, and the synthesized CDCF. For CNF, the peak at 1715 cm^−1^ indicates carboxylic acid groups [27,28], which originated from the selective TEMPO oxidation of the hydroxyl group at the C-6 position in the glucose ring [29]. After the polymerization reaction, new CDCF peaks emerged at 2932, 1261, and 1058 cm^−1^. The characteristic peaks at 2932 cm^−1^, 1261 cm^−1^, 1058 cm^−1^, and 3446 cm^−1^ are caused by the C-H stretching vibration of the methyl group, C-C stretching vibration [30], C-O-C stretching vibration, and -NH stretching vibration, respectively [31]. This indicates that the crosslinking reaction between CNF and BMA occurred, since, in this experiment, CDCF composites were prepared using free radical copolymerization.

**Figure 3:**
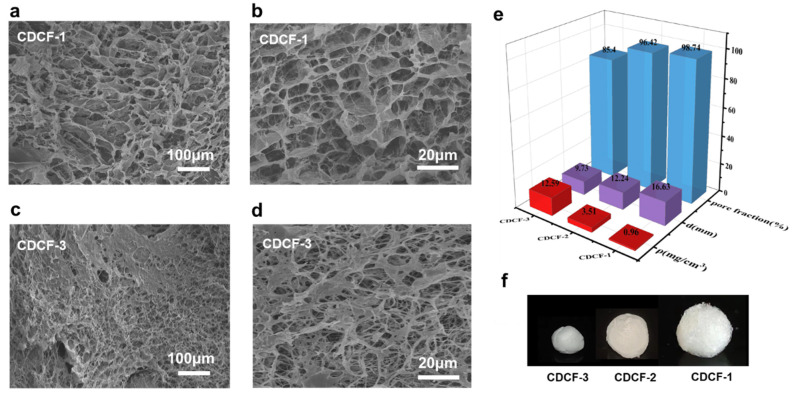
SEM photographs at different magnifications of (**a**,**b**) CDCF with 0.25 wt% BMA. (**c**,**d**) CDCF with 1 wt% BMA. (**e**) Pore fraction, size, and density properties of CDCFs with different numbers of crosslinkers. (**f**) Digital camera picture of CDCFs with different numbers of crosslinkers.

XPS tests were performed on polymerized samples before and after washing, as shown in Figure 2b. The resulting N_1s_ peaks were found in both samples, which proved that the covalent crosslinking caused by BMA was successfully achieved in CDCF and was not eluted after the samples were washed until neutral (pH = 7). In this situation, the covalent crosslink maintained its network structure after the cleavage of hydrogen bonding. Figure 2c,d show the TGA and DTG curves of CDCF-1 and CDCF-3. In the first stage between 0 and 150 °C, the mass of several samples decreased by approximately 5% due to residual water. A significant thermal degradation was observed between 210 °C and 450 °C, with a mass loss of approximately 60%, which suggested complicated processes, including the dehydration of the breaking of the C–O–C glycosidic bonds and the formation of anhydride, main-chain scission, destruction of the crosslinked network structure, and the breakage of PAA chains. However, the maximum weight loss of CDCF-1 (351 °C) was much faster than that of CDCF-3 (422 °C), according to the DTG curves. It was speculated that the expansion rate, porosity, and density increased in the CDCF samples with lower levels of BMA crosslinkers (as shown in Figure 3), thus favoring heat conduction in polymer networks [32].

The physical properties of CDCF samples with different numbers of covalent crosslinkers, including morphology, porosity, density, and expansion rate, are shown in Figure 3. The morphological evaluation of the CDCF samples is shown in Figure 3a–d. All samples have honeycomb-like porous structures in which CNF nanofibers interpenetrated around the pores. These structures favor vapor penetration into the polymer network, i.e., when CDCF is immersed in the vapors, these can easily diffuse into CDCF through the micropores. The pore size of the CDCF samples varied from several to tens of micrometers, as shown in the SEM images. Upon comparing two samples of CDCF-1 and CDCF-3, the pore size of CDCF-1 was much larger than that of CDCF-3. This is because the crosslinking density of CDCF-3 is much larger than that of CDCF-1. The diameter, density, and porosity values of CDCF-1, CDCF-2, and CDCF-3 are listed in Figure 3e. With varying numbers of crosslinkers, the density of the aerogels varied between 0.96 and 12.59 mg/cm^3^ and porosity decreased from 98.74 to 85.4%, corresponding to the volume expansion rate of different samples (Figure 3f). It should be noted that the minimum addition of a crosslinker is 0.25 wt%. Below this value, a ball will not form after washing treatment.

**Figure 4:**
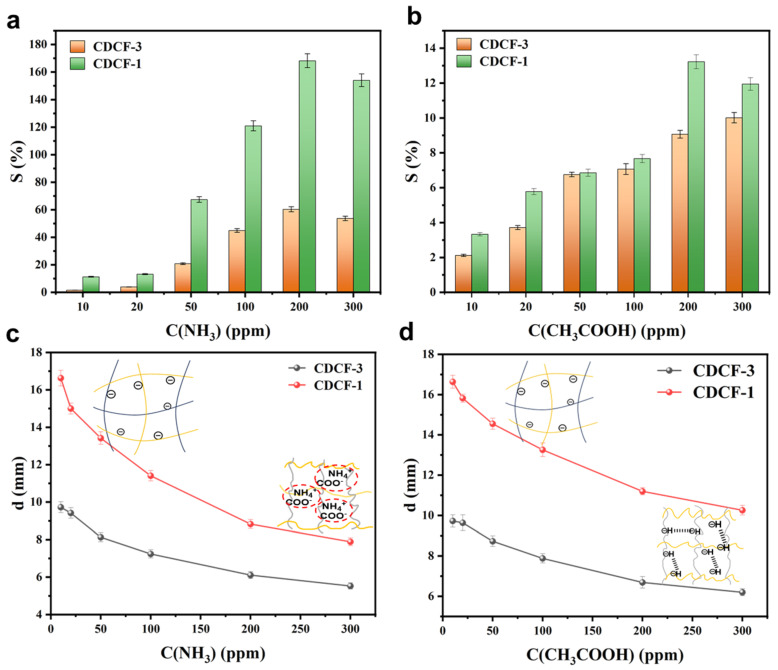
Vapor sensitivity of CDCF-1 and CDCF-3 as detected in the current analysis under different (**a**) NH_3_ and (**b**) CH_3_COOH concentrations. Vapor sensitivity of CDCF-1 and CDCF-3 detected in the size changes under different (**c**) NH_3_ and (**d**) CH_3_COOH concentrations. The inserts are mechanism diagrams of polymer chain interactions under different vapors using different (**a**) NH_3_ and (**b**) CH_3_COOH concentrations.

Figure 4 shows the sensitivities of CDCF-1 in ammonia and acetic acid vapors. Changes in sensitivity alongside shape and current changes were detected by placing CDCF-1 and CDCF-3 samples in an enclosed space filled with ammonia and acetic acid vapors, as shown in Figure S2. From Figure 4a–c, it is clear that acid/alkaline vapors have a significant impact on the shape and current sensitivity of CDCF-1 and CDCF-3. In contrast, CDCF experienced little change in neutral water vapor (Figure S3). The sensitivity mechanism is believed to occur in acetic acid vapors, and -COO^−^ in the CDCF chain protonates to -COOH, which weakens the repulsion between polymer chains and causes a decrease in CDCF (Figure 4c, insert) [33]. However, in ammonia vapor, NH^4+^generated by the dissociation of NH_3_ can more easily attach to -COO^−^ through coulomb forces, weakening the repulsive force between anions and polymer chains, thus causing a decrease in CDCF (Figure 4d, insert) [34]. This is in contrast to samples exposed to pure water vapors (pH = 7), which only decreased by 7% in diameter (Figure S3).

When the ammonia/acetic acid concentration increased from 10 to 200 ppm, the sensitivity of both samples sharply increased. Contrastingly, with the increase in vapor concentration from 200 ppm to 300 ppm, the sensitivity of both samples slightly decreased due to the saturated adsorption of ammonia and acetic acid at a concentration of 200 ppm. Moreover, the sensitivities of the response of each sample to acetic acid are far lower than those of ammonia. This is because the hydrogen bonding force generated in acidic vapor is weaker than the coulomb force in alkaline vapor. When comparing the sensitive properties of CDCF-1 and CDCF-3, CDCF-1 shows a much more sensitive performance regardless of whether ammonia or acetic acid vapor is used, indicating that the higher porosity structure of CDCF-1 favors vapors that penetrate into the structure, thus contributing to sensitivity.

**Figure 5:**
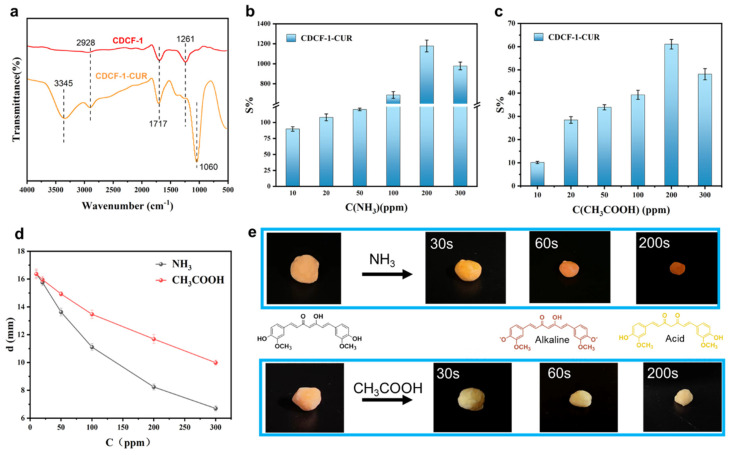
(**a**) FTIR spectra of CDCF-1 and curcumin-incorporated CDCF-1 (CDCF-1-CUR). (**b**) Vapor sensitivity of CDCF-1-CUR detected in the current analysis under different (**b**) NH_3_ and (**c**) CH_3_COOH concentrations. (**d**) Vapor sensitivity of CDCF-1-CUR detected by monitoring size changes under different NH_3_ and CH_3_COOH concentrations. (**e**) Color and size changes of CDCF-1-CUR under NH_3_ and CH_3_COOH vapors, for which the concentration was 300 ppm.

In order to improve the recognition diversity of CDCF, curcumin (CUR), a natural dye with color sensitivity to different pH values, was used [35]. The FTIR characteristic peak of CDCF-1-CUR at 1060 cm^−1^ belongs to the stretching vibration of the ether group, and the characteristic peak at 2926 cm^−1^ belongs to the symmetric stretching vibration of the methylene group [36], indicating that curcumin was successfully incorporated into CDCF-1 (Figure 5a). The sensitivity of CDCF-1-CUR in ammonia and acetic acid vapors was also studied. As shown in Figure 5b, the current sensitivity value of CDCF-1-CUR reaches 1178%, and the diameter shrinks from 16.37 mm to 7.24 mm in ammonia vapor at a concentration of 200 ppm. From Figure 5c, the concentration is 200 ppm, the current sensitivity value of CDCF-1-CUR is 61.06%, and the diameter shrinks from 12.61 mm to 6.34 mm in a 200 ppm concentration of acetic acid vapor. Both values are better than those obtained using individual CDCF-1, indicating that the incorporation of curcumin can improve sensitivity. Moreover, CDCF-1-CUR can change color in different ammonia and acetic acid vapors (Figure 5e). When placed in ammonia gas (300 ppm), the color of CDCF-1-CUR gradually changed from orange to reddish brown over a period of up to 200 s. When placed in acetic acid (300 ppm), its color remarkably changed from orange to yellow in the same time period. This color-changing mechanism is caused by the keto enol tautomerism of curcumin, whose chemical structure is presented in the form of enol esters in alkaline conditions and ketones in acidic conditions (Figure 5e). Performance summaries of reported vapor sensors and CDCF-CUR are given in Table S1; the detection limit and response time show moderate levels. However, CDCF-CUR, exhibiting dual shape and color changes when exposed to acid/alkaline vapors, shows great potential for the multi-detection of ammonia and acetic acid vapors.

## 4. Conclusions

In this study, a series of CDCFs with dual-crosslinked frameworks were prepared using the in situ free radical polymerization of AA and BMA in CNF gels, as confirmed by FT-IR (Figure 2a). An XPS study (Figure 2b) showed that covalent crosslinking occurred and was maintained before and after the increase in volume. Porosity, expansion rate, and density were controlled by changing the crosslinkers (Figure 3e). CDCF-1, which has a lower covalent crosslinking degree, remarkably changes in shape in acetic acid (a 2.1-fold decrease in diameter) and ammonia vapors (a 1.5-fold decrease in diameter). This mechanism is probably due to the reconstruction of hydrogen bonding in such vapors (Figure 4c,d, insert). Moreover, curcumin-incorporated CDCF-1 demonstrated significant color changes, varying between acid and alkaline vapors (Figure 5e). This discovery opens the door to a new class of observable detection materials with regard to acid/alkaline vapors such as acetic acid and ammonia vapors.

## Data Availability

Data are contained within the article.

## Supplementary Materials

The following supporting information can be downloaded at: https://www.mdpi.com/article/10.3390/polym16111547/s1, Figure S1: The preparation procedure of CDCF through in-situ free radical polymerization of AA, BMA in CNF gels; Figure S2: The equipment and experimental illustration of current sensitivity test; Figure S3: The size change rate of CDCF-1 and CDCF-3 samples in CH_3_COOH, H_2_O and NH_3_ vapor samples respectively. The concentration of CH_3_COOH and NH_3_ is 300 ppm, the pH of H_2_O is 7. Table S1. Summary of vapor sensors and their performance.
