# Supplementary material for: A Cellulose-Based Dual-Crosslinked Framework with Sensitive Shape and Color Changes in Acid/Alkaline Vapors
Source: Polymers (Basel). 2024 May 30;16(11):1547. doi: 10.3390/polym16111547 (PMC11174363; doi:10.3390/polym16111547)
Supplement: Supplementary file 1 [file polymers-16-01547-s001.zip › polymers-3001342-supplementary.pdf]

Supporting information

A Cellulose-Based Dual-Crosslinked Framework with Sensitive Shape and Color Changes in Acid/Alkaline Vapors

Yuxin Sun, Xinye Qian, Yan Gou, Chunling Zheng * and Fang Zhang *

College of Food Science and Light Industry, Nanjing Tech University, Nanjing 211800, China; 202161119011@njtech.edu.cn (Y.S.)

* Correspondence: zhengchunling@njtech.edu.cn (C.Z.); zfygjy@163.com (F.Z.)

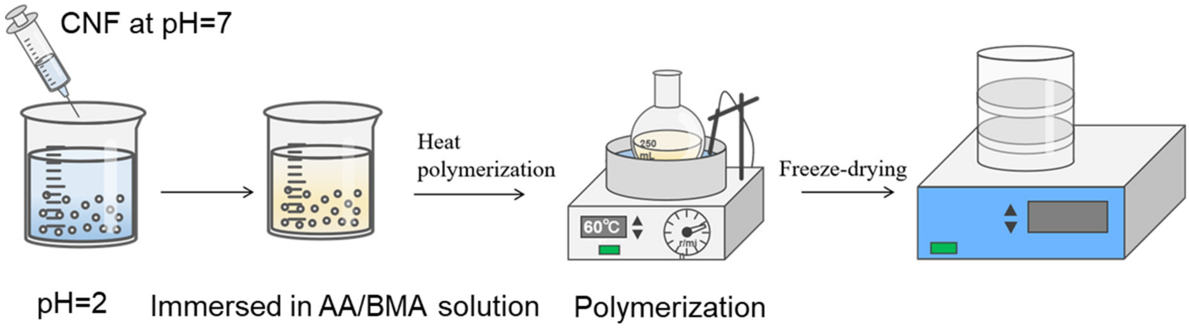

Figure S1 The preparation procedure of CDCF through in-situ free radical polymerization of AA, BMA in CNF gels.

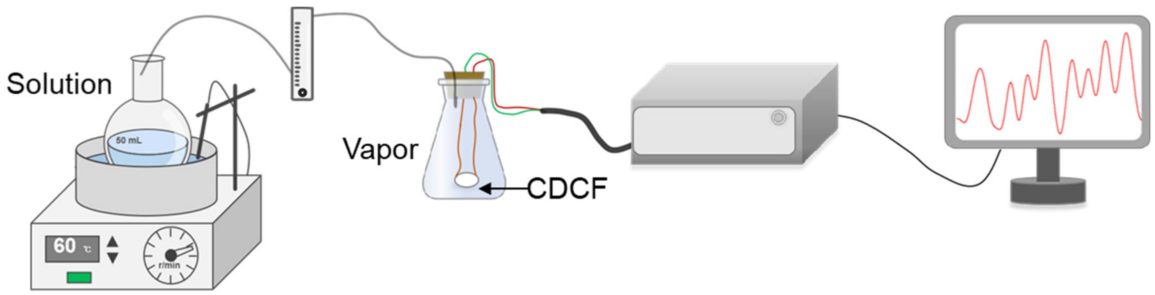

Figure S2 The equipment and experimental illustration of current sensitivity test.

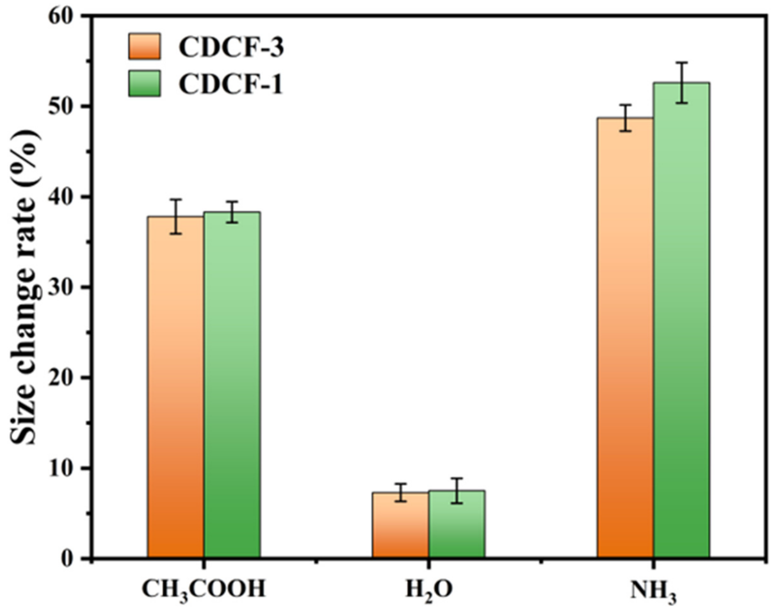

Figure S3 The size change rate of CDCF-1 and CDCF-3 samples in CH_3COOH, H_2O and NH_3 vapor samples respectively. The concentration of CH_3COOH and NH_3 is 300ppm, the pH of H_2O is 7.

Table S1. Summary of vapor sensors and their performance

Sensing Material	Analyte	Detection limit(ppm)	Response time(s)	Refs.
Copper(II) complex of the (Cu-PAN)	Ammonia	100	60	[1]

PPyNPs	Ammonia	0.1	19	[2]
ZnO/MWCNT	Ammonia	10	8.6	[3]
ZnO	acetic acid	100	50	[4]
Ag-LFO nanotubes	acetic acid	0.5	74.5	[5]
Mesoporous CuO	acetic acid	10	27.2	[6]
This work	Ammonia	7	3	
	acetic acid	10	16	

[1] L. Engel, I. Benito-Altamirano, K.R. Tarantik, C. Pannek, M. Dold, J.D. Prades, J. Wöllenstein, Printed sensor labels for colorimetric detection of ammonia, formaldehyde and hydrogen sulfide from the ambient air, Sensors and Actuators B: Chemical 330 (2021) 129281.

[2] W. Kim, S. Cho, J.S. Lee, Comparative Study on the Effect of Protonation Control for Resistive Gas Sensor Based on Close-Packed Polypyrrole Nanoparticles, Applied Sciences 10(5) (2020) 1850.

[3] L. Vatandoust, A. Habibi, H. Naghshara, S.M. Aref, Fabrication of ZnO-MWCNT nanocomposite sensor and investigation of its ammonia gas sensing properties at room temperature, Synthetic Metals 273 (2021) 116710.

[4] W.-F. Qin, H.-M. Zhang, X.-B. Li, Y.-W. Xing, Y.-X. Guo, Y.-Y. Feng, Y.-J. Li, S.-Q. Han, K.-F. Ma, H.-H. Cao, S.-N. Liu, X.-M. Jia, C.-H. Zhou, Surfactant modified hexagonal ZnO gas sensor for acetic acid, Journal of Materials Science: Materials in Electronics 34(20) (2023) 1560.

[5] J. Wu, Y. Wan, Z. Wang, Y. Wang, Q. Luo, C. Feng, T. Yoshinobu, Loose Ag-Doped $LaFeO_3$ Nanotubes-Based Gas Sensor for Excellent Acetic Acid Sensing, IEEE Sensors Journal 24(5) (2024) 5821-5829.

[6] W. Geng, Z. Ma, J. Yang, L. Duan, F. Li, Q. Zhang, Pore size dependent acetic acid gas sensing performance of mesoporous CuO, Sensors and Actuators B: Chemical 334 (2021) 129639.
